# Half-Yearly Report of Cases in Midwifery, Which Have Occurred in the Northern District of the London and Southwark Midwifery Institution

**Published:** 1832-02

**Authors:** C. Waller

**Affiliations:** Consulting Accoucheur to the above Institution, and Lecturer on Midwifery at 93, Bartholomew close.


### MIDWIFERY.

*Half-yearly Report of Cases in Midwifery, which have occurred in the Northern District of the London and Southwark Midwifery Institution.* By C. Waller, Esq. Consulting Accoucheur to the above Institution, and Lecturer on Midwifery at 93, Bartholomew close.

| 1831. | Number of Women delivered. | Sex of Children. | | Born Alive. | Stillborn. | Presentation. |
|---|---|---|---|---|---|---|
| | | Males. | Females. | | | |
| July ...... | 28 | 18 | 10 | 28 | 0 | 24 Natural<br>3 Breech<br>1 Face to Pubis |
| August ... | 43 | 24 | 20 | 41 | 3 | 43 Natural<br>1 Breech |
| September | 39 | 16 | 24 | 39 | 1 | 37 Natural<br>1 Breech<br>1 Foot<br>1 Face to Pubis |
| October... | 45 | 22 | 24 | 44 | 2 | 44 Natural<br>2 Breech |
| November | 48 | 27 | 21 | 47 | 1 | 46 Natural<br>1 Breech<br>1 Face to Pubis |
| December | 39 | 22 | 19 | 36 | 5 | 39 Natural<br>1 Foot<br>1 Face to Pubis |
| Total* ... | 242 | 129 | 118 | 235 | 12 | |

Several of the above, reported as Stillborn, were Premature Births.

Remarks. Several cases of interest have occurred during the last half-year, but want of time prevents my entering fully into their detail.

We have unfortunately had much disease in the institution, and have lost five patients: four died with that fell disease, puerperal fever, as it is called; in all, the cerebral symptoms were at the onset the most prominent; in two or

* Five women were delivered of twins; one in August, one in September, one in October, and two in December.

three instances out of the four, the abdominal symptoms were not developed until days had elapsed from the commencement of the attack, which I take for granted may, in every instance, be dated from the rigor, where this is followed by heat and flushing of the face, pain in the head, &c.

Many of the milder forms of this fever have also occurred, and in every case calomel and opium, with fomentations or counter-irritation, according to circumstances, have proved successful.

One case of active hysteritis has taken place, wherein recovery took place, though slowly. This patient was treated on the usual plan, viz. bleeding, mercurialization, and counter-irritation. I believe patients labouring under this disease, as well as acute inflammation of the peritoneum, frequently experience relapses, where the mercury has not been pushed to such an extent as to produce its well-known effect upon the salivary glands.

In one case the female had several times miscarried, and this event again took place between the sixth and seventh month. Her labour was marked by no peculiar symptom: she vomited, but this being so common, attracted no notice. After delivery, the vomiting continued, and, in spite of every effort, could not be checked. This poor woman died about the nineteenth day after her confinement, she experiencing no inconvenience except the vomiting. The remedies employed were leeching, blistering, calomel and opium, effervescing draughts, bluepill and opium, small doses of tartarised soda, &c., but without any mitigation of the symptoms. The matter ejected was generally discoloured, and mixed with a large quantity of mucous secretion, and every now and then there was an appearance of grumous blood mixed with it: the bowels acted very regularly. No post-mortem examination was instituted, and therefore the pathological condition of the mucous membrane of the stomach was not ascertained.

Two cases of convulsions have occurred; both of them happened to women in labour with their first child, and in both it was found necessary to deliver with instruments: one of them occurred during my absence in the country. This case was kindly superintended by my friend Dr. Steggall. The patient was a strong, robust Irish woman, and had been bled largely before the delivery was effected: the infant was born dead. In the other case, the first stage of the labour was remarkably slow, the os uteri beginning to dilate on the evening of the 23d of December: on the 24th I saw her, and found that dilatation was gradually progressing. On the morning of the 25th, the os uteri seemed open, but the pains feeble: a dose of the secale was administered, which increased the pains, but did not seem to advance the labour. In the course of a few hours, dilatation seeming to be complete, and there being plenty of secretion, I ruptured the membranes: after this, the pains greatly increased; the head descended into the pelvis until the parietal bones were nearly resting upon the perineum, but there it remained. As there was no particularly urgent symptom, I recommended patience, and left her under the care of my very intelligent pupil, Dr. Ogilvy, intending to call again in a couple of hours: I was, however, hastily summoned to the house, and found she had experienced a convulsive attack, and still seemed wandering. My friend and colleague, Mr. Doubleday, (whose opinion I had requested,) agreeing with me that it would not be proper longer to defer the delivery, I proceeded to introduce the forceps. There was not room to introduce them in the usual way, viz. one blade towards the pubis, the other towards the sacrum; I therefore introduced them laterally, and succeeded in getting a firm hold of the head, but, on attempting to draw down, the convulsions recurred, and I consequently desisted, and had recourse to the perforator. The patient had two convulsions afterwards during the day, and her head seemed very much confused; but she perfectly recovered. She was of a weakly and irritable habit; bloodletting, therefore, was not had recourse to.

One of the twin cases above reported happened at the fourth month of pregnancy: in this instance one of the fœtuses was of monstrous formation, there being a large spina bifida, and also a deficiency of the integuments of the abdomen: there was no diaphragm.

*Bartholomew Close; January* 1832.

